# Sliding to the rescue of damaged DNA

**DOI:** 10.7554/eLife.00347

**Published:** 2012-12-13

**Authors:** Bryan Gibb, Eric C Greene

**Affiliations:** **Bryan Gibb** is at the Department of Biochemistry and Molecular Biophysics, and the Howard Hughes Medical Institute, Columbia University, New York, United Statesbg2390@columbia.edu; **Eric C Greene** is at the Department of Biochemistry and Molecular Biophysics, and the Howard Hughes Medical Institute, Columbia University, New York, United Statesecg2108@columbia.edu

**Keywords:** Single Molecule, FRET, DNA repair, Homologous Recombination, E. coli

## Abstract

Single-molecule imaging experiments have shed new light on the methods used by the enzyme RecA to align single- and double-stranded DNA so that double-strand breaks can be repaired.

**Related research article** Ragunathan K, Liu C, Ha T. 2012. RecA filament sliding on DNA facilitates homology search. *eLife* **1**:e00067. doi: 10.7554/eLife.00067

**Image** Fluorescence data showing the RecA filament sliding on double-stranded DNA

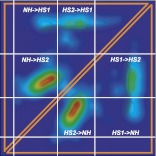

The homologous recombination of DNA—the exchange of stretches of DNA between two similar DNA molecules—is central to the repair of various types of DNA damage, and is also a real-life example of ‘searching for a needle in a haystack’. How does the recombination machinery inside cells bring together and align a short stretch of single-stranded DNA with an identical sequence of double-stranded DNA located within a genome that is comprised of millions or even billions of base pairs? Writing in *eLife*, researchers at the University of Illinois at Urbana-Champaign present evidence to suggest that a protein called RecA can help the single-stranded DNA slide along the double-stranded DNA until the molecules are properly aligned (Ragunathan et al., 2012).

Homologous recombination is a complex, yet highly conserved pathway that is required for the repair of DNA double strand breaks (Cromie et al., 2001) and replication forks that have stalled (Cox et al., 2000), and it also provides a means of generating genetic diversity. The importance of this repair pathway is revealed by the fact that double strand breaks lead to chromosomal abnormalities that are a hallmark of cancer.

Ever since it was discovered in 1965, the prokaryotic enzyme RecA has been a model system for scientists working on DNA repair. During homologous recombination, RecA binds to the single-stranded DNA that is generated at the double-strand break and forms a complex known as the pre-synaptic filament. This complex must somehow then survey the genome to find a matching double-stranded DNA partner through a process referred to as the ‘homology search’ (Figure 1). Once homologous DNA is found and the sequences are aligned, the pre-synaptic filament invades the duplex, ultimately allowing the DNA break to be repaired using the homologous DNA as a template to ensure fidelity.

**Figure 1.**
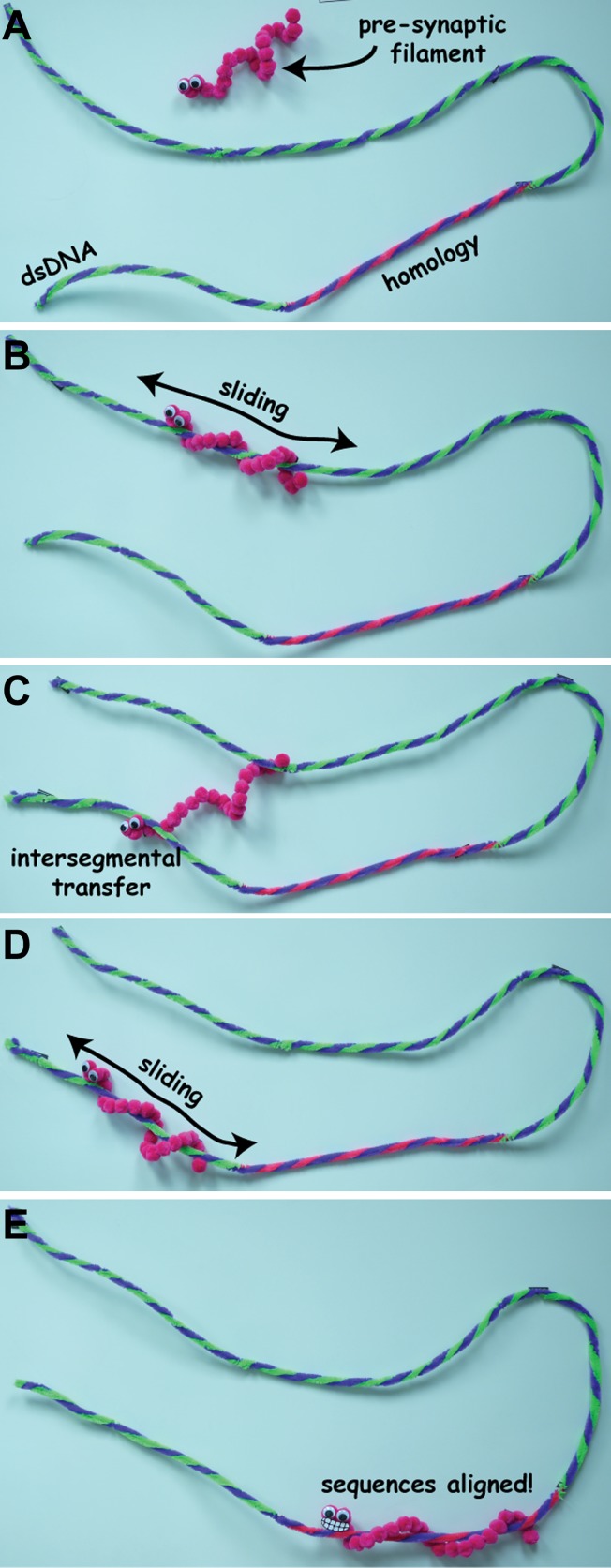
Stills from a video animation illustrating homology search (not to scale). The pre-synaptic filament has to find a specific sequence of bases (shown here in blue and pink and labelled ‘homology’) on a long molecule of double-stranded DNA (dsDNA). It initially binds to the dsDNA through 3D diffusion (**A**), and then slides back and forth along the dsDNA as it search for the sequence (**B**). If it cannot find the sequence, the pre-synaptic filament can move to a new location through intersegmental transfer (**C**), and start sliding back and forth along the dsDNA molecule again (**D**), until it finds the sequence of bases it is looking for (**E**). The full video is available at http://youtu.be/BmSrjVLb94c.

IMAGES: MYLES MARSHALL, BRYAN GIBB.

Herein lies the mystery: Exactly how does RecA find the correct stretch of DNA? Early work in the mid-1980s provided the first tantalizing hints that the homology search might be aided by a mechanism called intersegmental transfer that allows the pre-synaptic filament to, in effect, take a short cut by moving between distal regions of the double-stranded DNA (Gonda and Radding, 1986; Figure 1C).

In the late 1990s, however, another study concluded that the pre-synaptic filament did not slide along DNA: rather, this work suggested, the search mechanism was primarily mediated by three-dimensional (3D) diffusion (Adzuma, 1998). And earlier this year, researchers at UC Davis reported the results of single-molecule experiments which showed that the pre-synaptic filament was able to locate regions of homology when the duplex DNA was allowed to relax: however, the search was inhibited when the duplex DNA was stretched out (Forget and Kowalczykowski, 2012). These experiments convincingly demonstrated that intersegmental transfer does in fact contribute to the homology search, and that the pre-synaptic filament can simultaneously sample and move between different regions of double-stranded DNA that are far apart from one another. But this is not the end of the story.

In an effort to understand the homology search in even greater detail, the Illinois team—Kaushik Ragunathan, Cheng Liu and Taekjip Ha—have used single-molecule fluorescence resonance energy transfer (FRET) to explore if the pre-synaptic filament can slide along double-stranded DNA (Ragunathan et al., 2012). Single-molecule FRET is basically a ‘molecular ruler’ that can measure the distance between two different molecules if each is labelled with a small dye molecule that emits fluorescent light. In particular, it can measure very small changes in distance, on the order of just one billionth of a metre, so it offers much higher spatial resolution than has been achieved previously.

The Illinois team attached the pre-synaptic filaments to the surface of microscope slide and then watched in real-time as they searched for homology on short fragments of double-stranded DNA. The FRET signals fluctuated up and down, indicating that the pre-synaptic filaments were bound to the double-stranded DNA, and also revealing that the distance between the ends of the molecules was rapidly changing. By varying the length of the single-stranded DNA, Ragunathan, Liu and Ha determined that these fluctuations were caused by the pre-synaptic filament sliding along the double-stranded DNA fragments.

To confirm that sliding was taking place, the researchers next re-designed the single-stranded DNA sequence such that it could align with the double-stranded DNA at just one of two possible homologous locations, but not both at the same time. This allowed them to determine exactly how they were aligned at any given instant. As anticipated, they observed that the DNA molecules were aligned at one or other of the two homologous locations but, surprisingly, the Illinois team was also able to detect transitions between the two locations. Careful analysis of these transitions revealed that the pre-synaptic filaments could shuttle back and forth between the two regions of homology by sliding along the double-stranded DNA.

At first glance it might appear that this evidence for 1D sliding contradicts the intersegmental transfer mechanism reported by the UC Davis group. However, the two mechanisms are not incompatible, and it seems likely that both of these search modes are involved in homologous recombination. One possibility is that intersegmental transfer allows the pre-synaptic filament to sample different regions of double-stranded DNA over long distances, whereas 1D sliding is used for searching over much shorter distances (Figure 1).

The work of Ragunathan, Liu and Ha represents a crucial advance in our understanding of the molecular events that take place during the homology search. However, many important questions remain unanswered. For instance, how does the pre-synaptic filament actually sample the sequence of the double-stranded DNA to identify regions of homology? What are the relative contributions of 1D sliding and intersegmental transfer? How often must the pre-synaptic filament sample a region of homology before successful alignment can take place? Will eukaryotic proteins use similar mechanisms to align DNA sequences? How do the other proteins that are involved in homologous recombination influence the search for homology? And last but not least, how are searches for matching sequences carried out in the more complex physiological settings found in living cells? State-of-the-art single-molecule imaging technologies, such as those used by the Illinois team, are ready to start answering some of these questions.
